# Quantifying bunch-mode influence on photon-counting detectors at SPring-8

**DOI:** 10.1107/S1600577524001085

**Published:** 2024-02-16

**Authors:** Yasuhiko Imai, Takaki Hatsui

**Affiliations:** a Japan Synchrotron Radiation Research Institute, 1-1-1 Kouto, Sayo-cho, Sayo-gun, Hyogo 679-5198, Japan; b RIKEN SPring-8 Center, 1-1-1 Kouto, Sayo-cho, Sayo-gun, Hyogo 679-5148, Japan; Paul Scherrer Institut, Switzerland

**Keywords:** photon-counting 2D detectors, count-loss, bunch-modes, maximun count rates, rate correction

## Abstract

Count-loss characteristics of photon-counting 2D detectors are demonstrated for eight bunch-modes at SPring-8 through Monte Carlo simulations. As an indicator, the effective maximum count rate was introduced to signify the X-ray intensity that the detector can count with a linearity of 1% or better after applying a count-loss correction in each bunch-mode. The effective maximum count rate is revealed to vary depending on the bunch-mode and the intrinsic dead time of the detectors, ranging from 0.012 to 0.916 Mcps (megacounts per second) for a 120 ns dead time, 0.009 to 0.807 Mcps for a 0.5 µs dead time and 0.020 to 0.273 Mcps for a 3 µs intrinsic detector dead time. Even with equal-interval bunch-modes at SPring-8, the effective maximum count rate does not exceed 1 Mcps pixel^−1^. In other words, to obtain data with a linearity better than 1%, the maximum intensity of X-rays entering the detector should be reduced to 1 Mcps pixel^−1^ or less, and, in some cases, even lower, depending on the bunch-mode. When applying count-loss correction using optimized dead times tailored to each bunch-mode, the effective maximum count rate exceeds the values above. However, differences in the effective maximum count rate due to bunch-modes persist. Users of photon-counting 2D detectors are encouraged to familiarize themselves with the count-loss characteristics dependent on bunch-mode, and to conduct experiments accordingly. In addition, when designing the time structure of bunch-modes at synchrotron radiation facilities, it is essential to take into account the impact on experiments using photon-counting 2D detectors.

## Abstract 2

The effects of SPring-8 bunch-modes on the count-loss features of photon-counting detectors and effective maximum count rates are discussed.

## 1. Introduction

SPring-8 has proposed an upgrade to SPring-8-II in the latter half of the 2020s (RIKEN, 2014 ▸). Following this upgrade, experiments utilizing X-ray diffraction or scattering in the energy range 50–100 keV will become one of the most crucial research endeavors. For the measurement of X-ray diffraction or scattering in this energy range, hybrid photon-counting detectors with CdTe sensors have already been implemented. However, photon-counting detectors face difficulty in measurements at high count rates exceeding a few megacounts per second per pixel (Denes & Schmitt, 2014 ▸). Furthermore, CdTe, one of the sensor materials for high-energy X-rays, suffers from low hole mobility. To address this CdTe limitation, high-flux cadmium–zinc–telluride (CZT) has been developed, and research and development for its application in synchrotron radiation research are ongoing (Thomas *et al.*, 2017 ▸). Additionally, efforts have been made in developing integration-type detectors for synchrotron radiation experiments capable of exceeding the count rate limit of photon-counting detectors. One can find deployment examples in detectors such as JUNGFRAU (Leonarski *et al.*, 2020 ▸) and CITIUS (Grimes *et al.*, 2023 ▸; Takahashi *et al.*, 2023 ▸). Further improvements are now under investigation (Gadkari *et al.*, 2022 ▸; Fajardo *et al.*, 2020 ▸; Marras *et al.*, 2023 ▸). Given that the practical application of charge-integrating detectors tailored for high-energy X-rays in the energy range 50–100 keV is expected to take more time, the immediate utilization of photon-counting CdTe detectors or photon-counting high-flux CZT detectors is anticipated. The limitation of photon-counting detectors in the high count rate regime arises from the non-linearity of counting rates due to pile-up, which results in count-loss. It is known that this non-linearity depends on the bunch-mode (Bateman, 2000 ▸; Sobott *et al.*, 2013 ▸). Since the various bunch-modes at SPring-8 have been primarily designed to optimize experiments utilizing the time structure of synchrotron radiation, the adverse impact on the effective maximum counting rate of photon-counting detectors has become evident.

Therefore, in determining the bunch-modes for SPring-8-II, or any similar light source, it is imperative to consider not only the efficiency of experiments requiring the time structure but also the trade-off of deteriorated effective maximum count rates due to the pile-up-induced non-linearity of photon-counting detectors. Hence, we quantitatively evaluated how the effective maximum counting rate of photon-counting detectors is influenced by the bunch-modes at SPring-8 through Monte Carlo simulations.

## 2. Count-loss and correction

A photon-counting detector operates by electronically counting charge pulses in response to X-ray irradiation on its sensor. The electronic circuit, however, has a certain dead time during which it cannot distinguish two charge pulses generated by successive X-ray photons. This leads to pile-up events and count-loss, summarizing the simplified count-loss principle. Consequently, photon-counting detectors exhibit count-losses in regions of high-count-rate, necessitating correction when experiments such as high-resolution single-crystal diffraction measurements for charge-density studies require linearity in these high-count-rate regions.

The count-loss correction is fundamentally a straightforward calculation utilizing an effective dead time measured for each specific bunch-mode. At SPring-8’s X-ray absorption fine structure (XAFS) beamlines, for instance, count-loss corrections involving effective dead times are conducted for silicon drift detectors or germanium solid-state detectors, as XAFS experiments necessitate high linearity in data.

The incoming count rate (ICR), representing the number of incident X-ray photons per second on the detector, and the output count rate (OCR), denoting the number of X-ray photons counted by the detector per second, are parameters used for characterizing the count-loss features of detectors. A typical approach to measuring dead time involves varying the intensity of incident X-rays on the detector using multiple attenuators, and then recording the OCR against different levels of ICR. The dead time is derived by fitting the measurement results using a formula assuming either a paralyzable or non-paralyzable model, contingent on the detector system. These models are mathematically represented as equations (1) or (2), respectively (Knoll, 2000 ▸), 

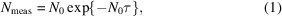




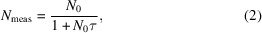

where *N*_meas_ is the measured count rate, *N*_0_ is the incoming count rate and τ is the dead time. Attention must be paid to potential higher harmonic components when employing an attenuator for synchrotron radiation X-rays. Reducing intensity using the attenuator leads to an increased proportion of higher harmonic components relative to the fundamental, given that the higher harmonics are less attenuated.

In the paralyzable model, an X-ray photon is counted if the time gap between the previous and subsequent X-ray photons exceeds the dead time. In this model, whether or not the immediately preceding incident X-ray photon has been counted does not affect the counting of the next photon. Conversely, in the non-paralyzable model, an X-ray photon is counted if the time interval between the last counted X-ray photon and the incoming X-ray photon of interest exceeds the dead time. Under the condition τ*N*_0_ ≪ 1, where the count rate is low, both models can be approximated by equation (3), 

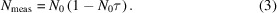

For our simulation, we employed the paralyzable model.

Since the detector response depends on the time structure of synchrotron radiation X-rays, dead time varies with bunch-modes. Consequently, for accurate count-loss correction, it is imperative to utilize the dead time measured under the corresponding bunch-mode for the specific experiment. Here, we define the dead time specific to a particular bunch-mode as the effective dead time (EDT), contingent on the specific bunch-mode. In contrast, the dead time measured when X-rays arrive at random timing is designated as the intrinsic dead time (IDT).

Count-loss correction functionality implemented in commercially available 2D detectors presumes random timing of incident X-rays, based on dead time measurements using laboratory X-ray generators, which exhibit random X-ray timing. Consequently, the count-loss correction functionality in commercially available 2D detectors may be less effective for high count rates with synchrotron X-rays, characterized by pulsed and unequally spaced time structures (Kraft *et al.*, 2009 ▸).

Furthermore, due to variations in characteristics among pixels of photon-counting 2D detectors, calibrations are performed to ensure these differences remain within an acceptable range. Notably, dead time also exhibits fluctuations from pixel to pixel. As such, measuring EDTs for every pixel across all bunch-modes to optimize count-loss correction for each individual pixel is impractical. This process entails translational scanning of the 2D detector within the detector window concerning X-rays, which is time-consuming, even with synchrotron radiation X-rays.

## 3. Simulation methods

Simulations were conducted for virtual detectors with IDTs of 120 ns, 0.5 µs and 3 µs, corresponding to typical dead times for the EIGER X 1M (DECTRIS AG), LAMBDA 750k (X-Spectrum GmbH) and HyPix-3000 (Rigaku Corp.) detectors, respectively. Note that the actual dead time varies based on factors such as X-ray energy, gain setting, threshold setting and the time structure of X-rays. For the simulations, the following assumptions were made.

(i) The detector response follows the paralyzable model, as expressed in equation (1).

(ii) The incident X-ray intensity is directly proportional to the charge of the electron bunch in the storage ring (*i.e.* the bunch current value).

(iii) Any decay in the stored ring current and temporal variations in X-ray intensity due to factors like the X-ray transport system, optics and other influences were dis­regarded.

(iv) The length of the electron bunch was neglected, assuming that all X-ray photons from one bunch reach the detector simultaneously.

The temporal length of the electron bunch is on the order of a few tens of picoseconds, significantly shorter than the dead time of the detector, allowing us to consider the X-rays to be arriving simultaneously.

The SPring-8 harmonic number (number of buckets) is 2436 (2 × 2 × 3 × 7 × 29) (Ego *et al.*, 1998 ▸). The time interval of the successive bunches (bucket interval) is 1.96625 ns. Thus, the orbital period is 1.96625 ns × 2436 = 4.789785 µs. One second is equivalent to approximately 208 778 orbits (1 s/4.789785 µs) of the SPring-8 storage ring. The OCR was obtained as the average of 100 one-second simulations with different seeds of random numbers. Specifically, for the (2436 × 208 778) buckets, the number of photons in the ICR were randomly assigned to buckets with the weight of the bunch current for each bucket. The timing of the X-ray photon incident on the detector is 1.96625 ns × the serial number of buckets. If the time difference between the previous X-ray photon and the next X-ray photon is longer than the IDT, the photon is counted; if the time difference is shorter, the photon is not counted.

There are eight bunch-modes at SPring-8: A-, B-, C-, D-, E-, F-, G- and H-modes, as shown in Fig. 3 in Appendix *A* [App appa] (RIKEN & JASRI, 2023 ▸). A-mode has an equal interval between bunches, and B- and C-modes are close to equal intervals. On the other hand, the bunch intervals in D-, E-, F-, G- and H-modes, called hybrid modes, are not equally spaced, but fillings consist of a train of continuous bunches and several isolated bunches with longer time intervals. The set values of the bunch current for each bucket of all bunch-modes are available online and can be downloaded as a CSV file (RIKEN & JASRI, 2023 ▸). Simulations were also performed for the complete multi-bunch (M-mode), in which all buckets are assumed to be equally occupied by electrons. In this M-mode, up to a certain count rate [*e.g.* 50 Mcps (megacounts per second)], X-rays can be regarded as incident on the detector at random timing. Thus, M-mode is the mode in which the IDT-based count-loss correction is anticipated to function accurately.

## 4. Results

**Figure 1:**
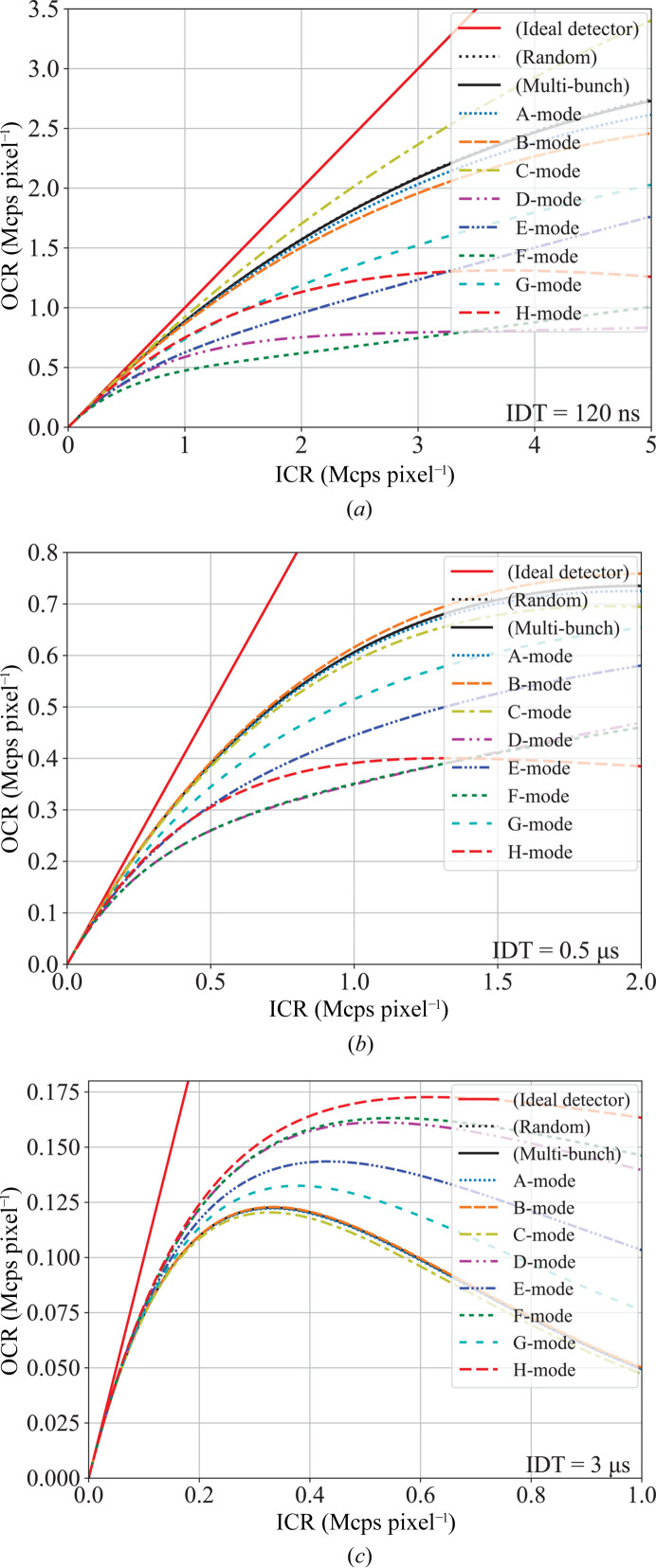
Response curves of virtual detectors with intrinsic dead times of (*a*) 120 ns, (*b*) 0.5 µs and (*c*) 3 µs to the incident X-ray intensity up to 5, 2 and 1 Mcps pixel^−1^ for SPring-8 bunch-modes A to H. The responses were calculated using Monte Carlo simulation based on the paralyzable model. The horizontal axis ICR represents the X-ray intensity per pixel incident on the detector, and the vertical axis OCR represents the count rate measured by the detector. Note that the EMCR, as defined in this study, are the values presented in Table 1 ▸.

Figs. 1 ▸(*a*), 1 ▸(*b*) and 1 ▸(*c*) show graphs of the OCR in terms of the ICR for SPring-8’s eight bunch-modes and M-mode, corresponding to detectors with IDTs of 120 ns, 0.5 µs and 3 µs, respectively. The black solid lines represent theoretical curves calculated using equation (1) with the respective IDTs. For an ideal detector without count-loss, the OCR is equal to the ICR, as shown by the solid red lines. However, count-loss results in the OCR being lower than the ICR. The degree of count-loss increases with higher counting rates. In Figs. 1 ▸(*a*) and 1 ▸(*b*), OCR curves for equally or near-equally spaced bunch-modes (A-, B- and C-modes) exhibit responses similar to the random incidence case. Conversely, OCR curves for hybrid modes (D-, E-, F-, G- and H-modes) show significantly lower responses compared with the random incidence case. In Fig. 1 ▸(*c*) with IDT = 3 µs, hybrid modes show higher responses in the high count rate range compared with equally spaced bunch-modes. This trend contrasts with IDT = 120 ns and 0.5 µs. In regions where the ICR is low, the difference in count-loss due to bunch-modes is not significant.

## 5. Discussion

Our simulations demonstrated variations in the OCR for the ICR across different bunch-modes at SPring-8 due to the differing count-loss behavior associated with each mode. Consequently, errors in results obtained through IDT-based count-loss correction also vary. To quantify the extent of count-loss correction achievable with acceptable errors, we introduced the concept of the effective maximum count rate (EMCR) as an indicator for each bunch-mode and detector.

**Figure 2:**
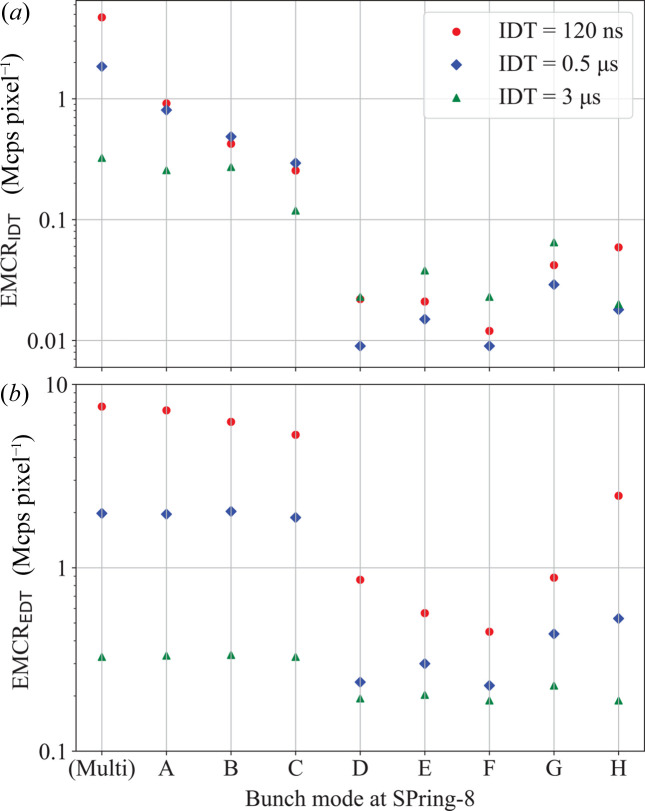
(*a*) EMCR_IDT_ and (*b*) EMCR_EDT_ for detectors with IDTs of 120 ns, 0.5 µs and 3 µs plotted as graphs. EMCR_IDT_ and EMCR_EDT_ are listed in Table 1 ▸. Multi indicates a complete multi-bunch-mode with a temporal interval of approximately 2 ns.

**Figure 3:**
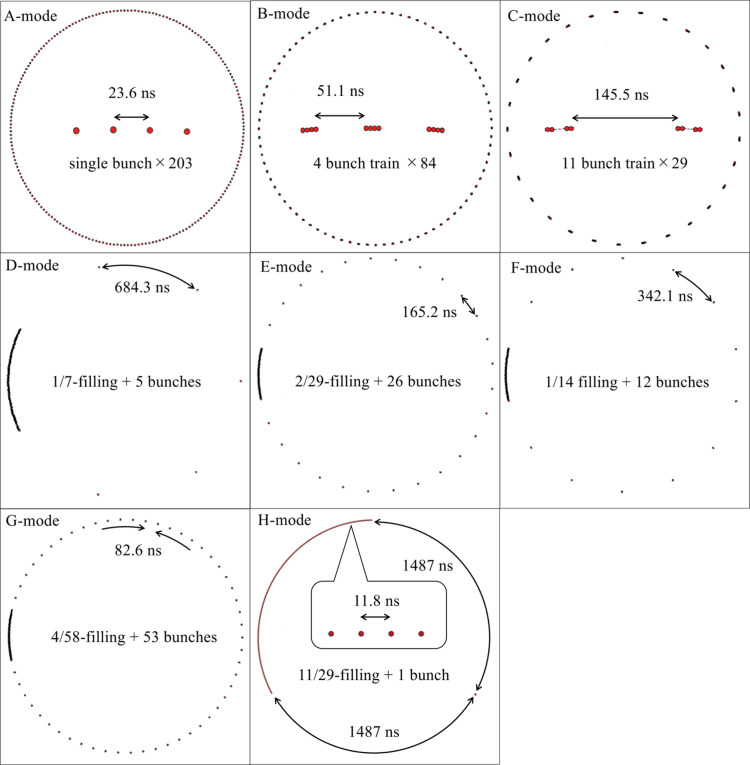
Schematics of the electron-filling patterns, bunch-modes, at SPring-8. The spacing between successive bunches in B- and C-modes and within the bunch train in D-, E-, F- and G-modes is approximately 2 ns.

EMCR_IDT_ and EMCR_EDT_ are defined as the maximum ICR at which the difference between the count rate obtained by count-loss correction with IDT or EDT and the OCR of an ideal detector with no count-loss does not exceed 1%. The count-loss correction was conducted by numerically solving the paralyzable model equation (1). The EDT for each bunch-mode was obtained by fitting the OCR as a function of the ICR using equation (1) with dead time τ as a parameter. The fitting range of the ICR extends from 0 Mcps pixel^−1^ to the ICR within this range where the maximum error, corrected with the EDT, becomes 1%. For example, considering Table 1 ▸ with IDT = 120 ns and D-mode, the range of the ICR used to obtain EDT = 542 ns is 0–0.860 Mcps pixel^−1^. Table 1 ▸ shows the EMCR_IDT_ and EMCR_EDT_ values for SPring-8 bunch-modes and detectors with three IDTs. To show clearly how much the EMCR_IDT_ and EMCR_EDT_ vary with different bunch-modes, graphs are shown in Fig. 2 ▸. It was found that EMCR_IDT_ values are remarkably low in D-, E-, F- and G-modes for IDT = 120 ns, limiting the performance of high-count-rate photon-counting 2D detectors even with their high counting rates. Similarly, for IDT = 0.5 µs and 3 µs, EMCR_IDT_ values remain significantly low in bunch-modes with non-uniform bunch intervals, such as D-, E-, F-, G- and H-modes.

For IDT = 120 ns, EMCR_IDT_ ranges widely from 0.916 Mcps pixel^−1^ for A-mode to 0.012 Mcps pixel^−1^ for F-mode, resulting in a factor of 76 difference. This implies that users conducting experiments during F-mode operation, where accuracy better than 1% is required, can only utilize X-rays with a maximum intensity attenuated to 1/76 of that in A-mode. This leads to a 76-fold difference in X-ray usage efficiency for users in F-mode operation. Even when count-loss correction is performed using EDTs optimized for the bunch-mode, EMCR_EDT_ exhibits large differences: 7.22 Mcps pixel^−1^ for A-mode and 0.448 Mcps pixel^−1^ for F-mode, a difference of 16 times. Although EMCR_EDT_ is larger than EMCR_IDT_, the difference due to bunch-mode remains substantial.

For IDT = 0.5 µs, the EMCR_IDT_ ranges from 0.807 Mcps pixel^−1^ for A-mode to 0.009 Mcps pixel^−1^ for D- and F-mode. This represents a 90-fold difference, which is larger than that observed for IDT = 120 ns. Even when corrected using EDT, the EMCR_EDT_ values are 2.03 Mcps pixel^−1^ in B-mode and 0.228 Mcps pixel^−1^ in F-mode, resulting in an approximately ninefold difference.

Similarly, for IDT = 3 µs, EMCR_EDT_ values are 0.273 Mcps pixel^−1^ for B-mode and 0.020 Mcps pixel^−1^ for H-mode, showing an approximate 14-fold difference. EMCR_EDT_ values are 0.335 Mcps pixel^−1^ for B-mode, and 0.189 Mcps for F- and H-mode, indicating a difference of about 1.8 times. Note that for the detector with IDT = 3 µs, count-loss correction using IDT for the hybrid modes would result in over-estimation. In the paralyzable model, under specific conditions, the OCR reaches its maximum value as seen in Fig. 1 ▸(*c*). Even with optimized correction models tailored to the IDT and bunch-mode, count-loss correction is applicable up to this maximum rate.

Fig. 1 ▸(*a*) shows that the C-mode OCR has a higher response compared with that of M-mode (complete multi-bunch). This is due to the fact that the train interval of C-mode is 145.5 ns, which is longer than the IDT of 120 ns. Consequently, up to one photon per train can be counted without any count-loss. However, this over-efficient counting for C-mode does not necessarily confer experimental advantage, except in specific cases. Applying a count-loss correction using the IDT to C-mode data would result in overestimation, which becomes problematic when experiments require high linearity in measured intensity. Furthermore, considering the general dispersion in dead time between pixels, there is a concern that this condition, IDT = 120 ns and a train interval of 144.5 ns, could lead to differences in count-loss between pixels, resulting in greater sensitivity dispersion. In order to reduce the effect of dead time dispersion, the bunch interval should be sufficiently shorter or longer than the dead time.

The bunch intervals for APS’s 24-bunch-mode and PETRA-III’s 40-bunch-mode are 153 ns and 192 ns, respectively, similar to SPring-8’s C-mode (145.5 ns). Therefore, for detectors with an IDT of 120 ns, correcting count rates using the IDT with the paralyzable model might overestimate the intensity.

The count-loss in the hybrid bunch-modes primarily occurs for X-rays emitted from the train section, where successive X-ray pulse intervals are shorter than the IDT. X-rays from the train sections exhibit a higher average temporal density within the timescale of the dead time, making the detector more susceptible to losing counts at a high count rate region. Note that, despite higher temporal density in isolated bunches, their contribution to count-loss is lower due to their relatively small percentage of the total stored current.

Detectors incorporating a retrigger functionality,^1^ designed to prevent paralysis and allow for higher count rates, have been developed (Trueb *et al.*, 2015 ▸). The EIGER2 and PILATUS3 detectors (DECTRIS AG), equipped with this feature, exhibit a response similar to the non-paralyzable model (Zambon, 2021 ▸). Simulations are conducted for the non-paralyzable model and results are presented in Appendix *B* [App appb]. It shows reduced count-loss at high rates, although the impact of bunch-mode differences persists.

This study relies on simulations assuming both paralyzable and non-paralyzable models. However, note that the response of the actual detector may not align with the simulation. Exploring the validation of simulation results through comparison with experimental data obtained using the real detector is an aspect to consider in the future.

## 6. Summary

The impact of SPring-8’s bunch-modes on count-loss in photon-counting detectors was quantitatively assessed through simulation. The results revealed that, for IDT = 120 ns, the EMCR in D-, E-, F- and G-modes is remarkably low (22, 21, 12 and 42 kcps pixel^−1^, respectively), imposing limitations on the performance of high-count-rate photon counting 2D detectors. These are modes in which the ring is partially filled with a bunch train and partially filled with widely spaced bunches. This limitation arises from count-loss correction functionalities assuming random timing of incident X-rays, which is not the case for synchrotron radiation X-rays with complicated time structures. When the bunch interval is close to the IDT, variations in counts between pixels due to dead time dispersion become significant. Thus, to mitigate pixel-to-pixel dead time dispersions, the bunch interval should substantially differ from the IDT. When considering the bunch-mode at synchrotron facilities, it is advisable to account for train width and bunch time intervals so that photon-counting detectors can be used effectively, together with the efficiency of experiments requiring X-ray time structures. Beamline scientists employing photon-counting detectors should also grasp the characteristics of these detectors and utilize them under conditions that meet the precision demands of experiments. For added convenience, a web application enabling easy simulation of count-loss for arbitrary bunch-modes is accessible online (Imai & Hatsui, 2024 ▸).

## Figures and Tables

**Figure 4:**
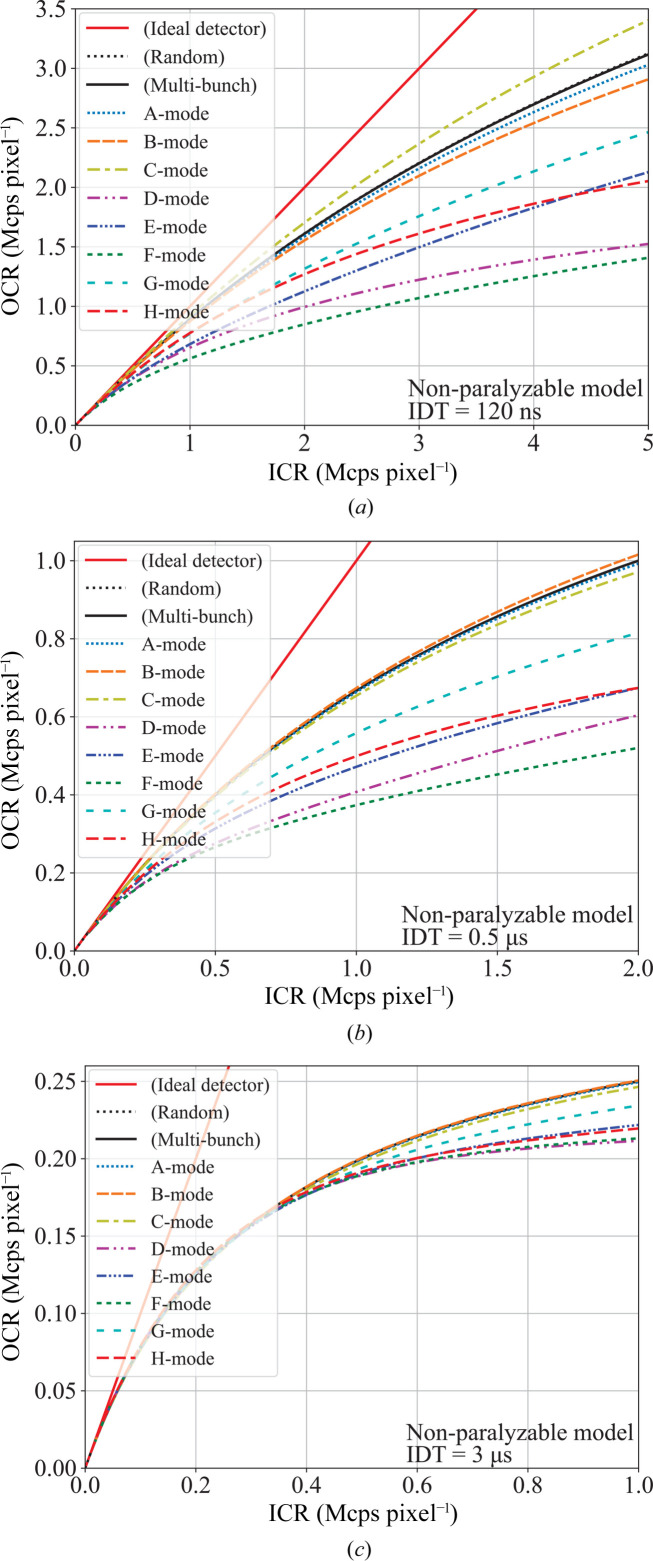
Response curves of virtual detectors with intrinsic dead times of (*a*) 120 ns, (*b*) 0.5 µs and (*c*) 3 µs to the incident X-ray intensity up to 5, 2 and 1 Mcps pixel^−1^ for SPring-8 bunch-modes A to H. The responses were calculated using Monte Carlo simulation based on the non-paralyzable model.

**Table 1:** Effective maximum count rate (EMCR) (Mcps pixel^−1^) of detectors with IDTs of 120 ns, 0.5 µs and 3 µs for SPring-8 bunch-modes EMCR_IDT_ and EMCR_EDT_ are defined as the maximum count rates at which the remaining error after the count-loss correction using the IDT or EDT is 1%. The EMCR values marked with an asterisk (*) indicate the intensity at which the OCR becomes maximal due to the count-loss, rather than the intensity at which the error becomes 1%. Beyond this maximum, count-loss correction becomes infeasible, making this point the practical maximum count rate. The values in parentheses represent the calculated EDTs obtained by fitting simulation results using equation (1).

	Effective maximum count rate (Mcps pixel^−1^)					
IDT	120 ns		0.5 µs		3 µs	
Bunch-mode	EMCR_IDT_	EMCR_EDT_	EMCR_IDT_	EMCR_EDT_	EMCR_IDT_	EMCR_EDT_
(Multi)	4.72	7.57* (121 ns)	1.85	1.98* (0.500 µs)	0.325	0.327 (3.00 µs)
A	0.916	7.22* (130 ns)	0.807	1.96* (0.507 µs)	0.257	0.332 (3.01 µs)
B	0.424	6.25 (142 ns)	0.486	2.03* (0.484 µs)	0.273	0.335 (2.99 µs)
C	0.255	5.32 (77.7 ns)	0.294	1.88 (0.529 µs)	0.119	0.327 (3.05 µs)
D	0.022	0.860 (542 ns)	0.009	0.238 (1.49 µs)	0.023	0.194 (2.51 µs)
E	0.021	0.566 (532 ns)	0.015	0.300 (1.07 µs)	0.038	0.203 (2.70 µs)
F	0.012	0.448 (854 ns)	0.009	0.228 (1.51 µs)	0.023	0.189 (2.51 µs)
G	0.042	0.883 (318 ns)	0.029	0.436 (0.769 µs)	0.065	0.228 (2.84 µs)
H	0.059	2.47 (285 ns)	0.018	0.529 (0.993 µs)	0.020	0.189 (2.42 µs)

**Table 2:** Bunch-mode configuration of SPring-8

Mode	Bunch configuration	Bunch interval (ns)
A-mode	203 bunches	23.6
B-mode	4-bunch train × 84	51.1
C-mode	11-bunch train × 29	145.5
D-mode	1/7-filling (85 mA) + 5 bunches (3 mA)	684.3
E-mode	2/29-filling (63.6 mA) + 26 bunches (1.4 mA)	165.2
F-mode	1/14-filling (80.8 mA) + 12 bunches (1.6 mA)	342.1
G-mode	4/58-filling (47 mA) + 53 bunches (1 mA)	82.6
H-mode	11/29-filling (95 mA) + 1 bunch (5 mA)	11.8, 1486

## Footnotes

1 EIGER (DECTRIS AG), LAMBDA (X-Spectrum GmbH) and HyPix-3000 (Rigaku Corporation) detectors do not support the retrigger functionality and are supposed to follow the paralyzable model.

## Appendix A Bunch-modes at SPring-8

Fig. 3 ▸ shows schematics of the electron-filling patterns and bunch-modes at SPring-8. There are eight patterns, namely A- to H-modes. A-modes consists of 203-bunches with equal intervals. B- and C-modes consist of 84 of 4-bunches or 29 of 11-bunches with equal intervals. D- to H-modes, on the other hand, are hybrid modes composed of a continuous bunch section and isolated bunches. The continuous bunch section is referred to as a train. A train is a series of bunches with an interval of approximately 2 ns, except for H-mode. In H-mode, the train consists of bunches with intervals of 11.8 ns. For each mode, the corresponding bunch configuration and intervals are summarized in Table 2 ▸. Additional details and information on the bunch-modes can be found on the relevant webpage (RIKEN & JASRI, 2023 ▸). Furthermore, the design values of electron current for every 2436 buckets are also open to the public.

## Appendix B Simulation results for the non-paralyzable model

The results of Monte Carlo simulations conducted under the assumption that the count-loss follows the non-paralyzable model are presented. Figs. 4 ▸(*a*), 4 ▸(*b*) and 4 ▸(*c*) show OCR curves for the SPring-8 bunch-modes corresponding to detectors with IDTs of 120 ns, 0.5 µs and 3 µs, respectively. Although the paralyzable model exhibited cases where the OCR decreased with an increase in the ICR due to detector paralysis under certain conditions, the non-paralyzable model consistently showed an increasing trend of the OCR with respect to the ICR. It can be observed that the response of the OCR with respect to the ICR in the non-paralyzable model significantly varies across bunch-modes, similar to that of the paralyzable model. OCR curves for the equally or quasi-equally spaced A-, B- and C-modes are close to that of the complete multi-bunch-mode, M-mode. On the other hand, OCR curves for the unequally spaced D-, E-, F-, G- and H-modes are lower than those for the equally spaced modes, resembling characteristics similar to the paralyzable model. Therefore, even for non-paralyzable, the degree of count-loss depends on the bunch-mode.
